# Supplementary material for: A set of multi-entry identification keys to African frugivorous flies (Diptera, Tephritidae)
Source: Zookeys. 2014 Jul 24;(428):97–108. doi: 10.3897/zookeys.428.7366 (PMC4143993; doi:10.3897/zookeys.428.7366)
Supplement: Supplementary material 6 — Key to Ceratitis [file zookeys-428-097-s006.zip › SF6_ZooKeys_key to Ceratitis/key/SF6_ZooKeys_key to Ceratitis/Media/Html/Ceratitis punctata.htm]

Ceratitis (Pardalaspis) punctata (Wiedemann) 


***Ceratitis*** **(*Pardalaspis*)
*punctata* (Wiedemann)**

*Tephritis* *punctata* Wiedemann, 1824: 55.

Body
length: 6.86 (6.0-7.5) mm; wing length: 6.86 (6.5-7.0) mm.

Male

Head.
Antennal segments orange-red. Third antennal segment twice as long as second
segment. Arista basal part orange coloured, otherwise dark. Frons yellowish in
ground colour, silvery over entire length; with dispersed short hairs,
distinctly darker than frons. Ocellar triangle dark. Face orange to orange-red;
no median band. Occiput moderately swollen below, pale whitish. Chaetotaxy
normal for subgenus.

Thorax.
Ground colour of mesonotum greyish to grey-brown; also with three poorly
defined darker stripes, usually interrupted; sometimes with darker spots along
prescutellar acrostichal setae. Postpronotum same colour as mesonotum.
Chaetotaxy normal for subgenus. Mesonotum with pale pilosity. Anepisternum
along upper margin with darker hairs, otherwise pale pilosity. Two anepisternal
bristles.� Scutellum yellow with apical markings black; basally with two
distinct dark spots.

Legs
dark yellow to orange brown; setation typical of subgenus. Setae dark, front
femur with posterior row dark.

Wings
with brownish bands, yellow markings strongly reduced. Banding, setation and
venation normal for subgenus. Marginal band continuous; discal band joined with
marginal band; cross-vein r-m at middle of discal cell; vein R_1_
ending before or oppostie cross-vein r-m.

Abdomen.
greyish, with clearly defined spots. Pattern of spots and setation normal for
subgenus.

Female

As
male except for the following characters. Third antennal segment two to three
times as long as second. Frons yellow, not silvery. Face wholly yellow; at
antennal socket sometimes with a darker horizontal band. Oviscape shorter than
abdominal terga 3-6, orange in colour.

(Description
after De Meyer, 1996)
